# Accuracy of Measuring Blood Pressure with a Volume Clamp-Based Finger Cuff vs. Arterial Line at Rest and During Exercise in Patients with Pulmonary Hypertension: A Post Hoc Analysis

**DOI:** 10.3390/jcm15083033

**Published:** 2026-04-16

**Authors:** Anna Titz, Julian Müller, Simon Raphael Schneider, Stéphanie Saxer, Esther Irene Schwarz, Mona Lichtblau, Silvia Ulrich

**Affiliations:** 1 Department of Pulmonology, University Hospital Zurich, 8091 Zurich, Switzerland; 2 Department of Health, Eastern Swiss University of Applied Sciences, 9007 St Gallen, Switzerland; 3 Faculty of Medicine, University of Zurich, 8032 Zurich, Switzerland

**Keywords:** continuous blood pressure measurement, volume-clamp method, finger cuff, Finapres, Bland–Altman, Taffé method, pulmonary hypertension, exercise

## Abstract

**Background/Objective**: Continuous blood pressure (BP) monitoring is essential in clinical settings, where rapid hemodynamic changes influence patient management. While intra-arterial measurement remains the reference standard, non-invasive volume-clamp systems offer a potential alternative. We assessed the accuracy of finger-cuff-based continuous BP monitoring compared to invasive measurement in patients with pulmonary hypertension (PH). **Methods**: This post hoc analysis from a crossover RCT included PH patients who underwent repetitive hemodynamic assessments at rest and during exercise. The participants had simultaneous invasive BP monitoring via the radial artery and a non-invasive finger-cuff device (Finapres^®^ NOVA Basic). The mean blood pressure (mBP) was compared at rest, 50% of the maximal workload, and at the end of exercise using Bland–Altman and Taffé analysis. **Results**: In the study, 24 patients (seven female; 59 ± 14 years) contributed 385 paired mBP measurements. The invasive and non-invasive methods showed similar values at rest (96.1 ± 16.7 vs. 96.4 ± 17.2 mmHg) and during maximal exercise (106.8 ± 18.6 vs. 111.8 ± 21.6 mmHg). The overall Bland–Altman bias was 2.8 mmHg with wide limits of agreement (−39.6 to 45.3 mmHg), which remained broad across all exercise intensities. The Taffé analysis revealed a non-uniform, directionally dependent bias: the non-invasive system overestimated the mBP at low pressures and underestimated it at higher pressures. The measurement variability was substantially greater for the non-invasive method than for the invasive reference. **Conclusions**: In PH patients, finger-cuff-based continuous BP monitoring demonstrated acceptable group-level agreement but insufficient individual-level accuracy for clinical decision-making.

## 1. Introduction

Blood pressure (BP) is by far the most commonly measured hemodynamic parameter. The intra-arterial BP measurement via an arterial cannula and a calibrated pressure transducer remains the clinical reference method for continuous measurement and is preferred over non-invasive methods when an exact measurement is required. However, in hemodynamically stable patients, valuable information can be obtained with non-invasive techniques [1].

Continuous BP measurement is valuable in clinical scenarios where rapid or frequent BP fluctuations may impact patient management or outcomes, such as critical care or during exercise [1]. During exercise, BP measurement is especially challenging due to movement and noise, but it provides important prognostic and diagnostic information. Abnormal responses, such as an exaggerated rise or a failure to increase systolic BP, may indicate underlying cardiovascular risk or dysfunction [2].

BP may be continuously measured invasively with an intra-arterial line. On the other hand, BP can also be continuously assessed non-invasively based on the volume-clamp method with a finger cuff. The cuff is placed around the finger, and the cuff pressure adjusts with a high frequency to maintain constant arterial volume in the finger, which is then monitored by photoplethysmography and allows for pulse wave analysis [3]. This method enables beat-to-beat BP monitoring and is implemented in devices such as Finapres^®^ NOVA. The American Heart Association describes the volume-clamp method as providing continuous arterial pressure waveforms, but notes that the reproducibility depends on factors such as cuff placement, finger position relative to the heart, and peripheral perfusion [1].

Clinical trials comparing finger-cuff volume-clamp devices to invasive BP measurement have demonstrated that finger-cuff systems can provide reasonable estimates of BP and track hemodynamic changes, but do not consistently meet the criteria for clinical interchangeability in critically ill patients. The meta-analyses and method-comparison studies show mean biases that are typically within 5 mmHg but with wide limits of agreement and significant drift over time, especially after cardiovascular interventions or in patients with poor peripheral perfusion [3,4,5,6,7].

Pulmonary hypertension (PH) encompasses a spectrum of pulmonary vascular diseases, with pulmonary arterial hypertension (PAH) and chronic thromboembolic pulmonary hypertension (CTEPH) representing key subtypes that are characterized by progressive pulmonary vascular remodeling, increased pulmonary vascular resistance, and eventual right ventricular failure [8]. In patients with PH, accurate and continuous BP assessments carry particular clinical weight. These patients frequently undergo right heart catheterization during exercise as part of a diagnostic workup or disease monitoring, during which systemic hemodynamic stability must be closely observed. Exertional hypotension or an attenuated BP response during exercise may signal impaired cardiac reserve and right ventricular arterial uncoupling and can directly influence clinical decision-making regarding treatment escalation or activity recommendations. At the same time, the peripheral circulatory abnormalities that are characteristic of PH—including reduced cardiac output, elevated systemic vascular resistance, and peripheral edema—may fundamentally alter the performance of non-invasive BP devices that rely on the assumptions of normal peripheral perfusion.

An accurate hemodynamic assessment at rest and during exercise is essential for diagnosis, risk stratification, and therapeutic monitoring in these conditions, but non-invasive continuous BP monitoring technologies, such as volume-clamp methods, are not yet validated in this patient population [9,10,11,12]. This raises the question of whether volume clamp-based finger-cuff systems, which have been evaluated in surgical and critically ill populations, remain valid in the context of PH, where systemic hemodynamics are governed by a very different pathophysiological substrate. Despite the clinical relevance of this question, no prior studies have specifically validated continuous non-invasive BP monitoring against an invasive reference standard in patients with PH during graded exercise. Therefore, the aim of the current analysis is to evaluate the accuracy of a cuff volume-clamp device compared to invasive BP measurement at rest and during exercise in patients with PH.

## 2. Materials and Methods

**Figure 1:**
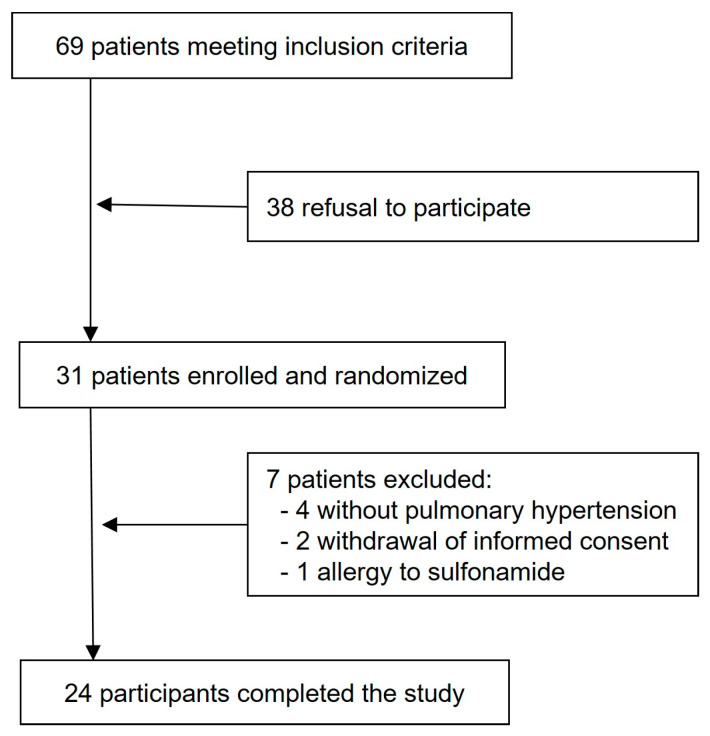
The study flow chart.

This is a post hoc analysis of data from a prospective, randomized, triple-phase, placebo-controlled, double-blinded crossover trial evaluating the acute hemodynamic effects of acetazolamide at rest and during exercise. The study design, methods, and results have been previously described, but the BP data have not been published before [13,14,15] (Figure 1). The patients with PAH or CTEPH were included, and written informed consent was obtained from all the participants. The study was conducted in accordance with the Declaration of Helsinki, received approval from the local ethics committee (BASEC 2016-00089), and was registered at ClinicalTrials.gov (NCT02755259).

The participants underwent right heart catheterization and completed cycling exercises in a semi-supine position using a protocol with stepwise increments of 10–20 Watts every 3 min until exhaustion (Thera-vital Ergometer; Medica Medizin GmbH, Lüdenscheid, Germany).

Systemic BP was assessed invasively through a 5F Teflon catheter placed into a radial artery and connected to a calibrated pressure transducer that was leveled at the phlebostatic axis and zeroed to atmospheric pressure. In addition, BP was measured non-invasively with the Finapres^®^ NOVA Basic device (Finapres Medical Systems, Enschede, The Netherlands) using the hemodynamics application module, which includes a finger cuff and an upper arm cuff for brachial calibration. All signals were recorded continuously and stored using LabChart (Version 8.1.16; ADInstruments, Dunedin, New Zealand). The collected data were visually reviewed to confirm plausibility, and the artifacts were removed.

The analysis primarily focused on the mean BP (mBP). Unless stated otherwise, the results were reported as mean ± SD unless indicated otherwise. The agreement between measurement techniques was evaluated using Bland–Altman analysis to determine the bias and limits of agreement; the Taffé analysis was applied to further examine the structure of the systematic bias, providing adjusted estimates of bias and proportional error across the range of mBP values. The statistical analysis was performed in R Version 4.5.0.

## 3. Results

### 3.1. Baseline Characteristics and Hemodynamic Measurements

A total of 24 patients (seven female) with PH were included in the study. The mean age was 59 ± 14 years, and the mean pulmonary artery pressure was 37 ± 11 mmHg. Seventeen patients (71%) were diagnosed with chronic thromboembolic PH, and seven (29%) had pulmonary arterial hypertension. The baseline demographic and hemodynamic data are summarized in Table 1.

### 3.2. Comparison Between Continuous BP Measurement Methods

At rest, the mean blood pressure was similar when measured invasively and non-invasively (96.1 ± 16.7 mmHg vs. 96.4 ± 17.2 mmHg). By the end of the exercise, the mean blood pressure increased with both methods, reaching 106.8 ± 18.6 mmHg invasively and 111.8 ± 21.6 mmHg non-invasively (Table 2).

Table 3 demonstrates the Bland–Altman analysis comparing the continuous non-invasive system with the reference method. The overall bias was 2.84 mmHg with wide limits of agreement (−39.62 to 45.31 mmHg). The agreement was highest at rest (bias 0.79 mmHg, LoA −37.15 to 38.73 mmHg) and remained consistent across the increasing workloads.

**Figure 2:**
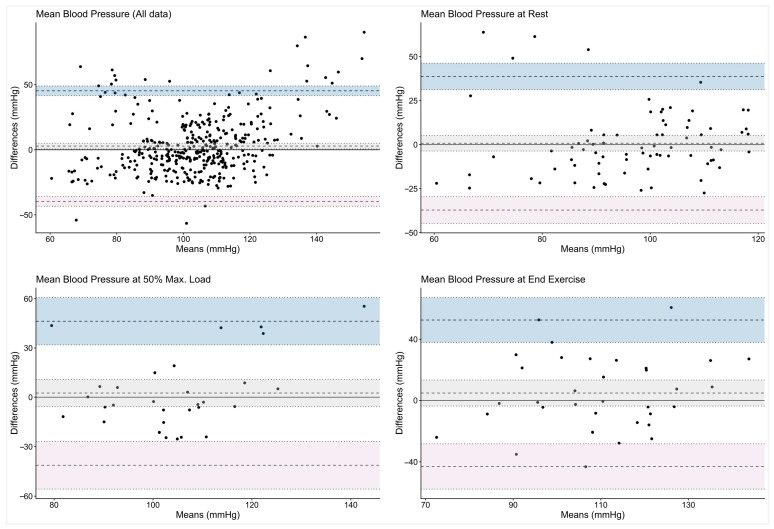
The Bland–Altman plots. The given symbols represent: **•** individual subjects; ── bias (mean difference); ╍╍╍ limits of agreement; and ┉┉┉ confidence interval for bias and limits of agreement.

The graphical analysis illustrates the wide dispersion of the mBP values irrespective of the workload. The Bland–Altman plots (Figure 2) show broad limits of agreement between the non-invasive and reference techniques, indicating substantial variability. The spread of data remains consistent, independent of the exercise intensity.

**Figure 3:**
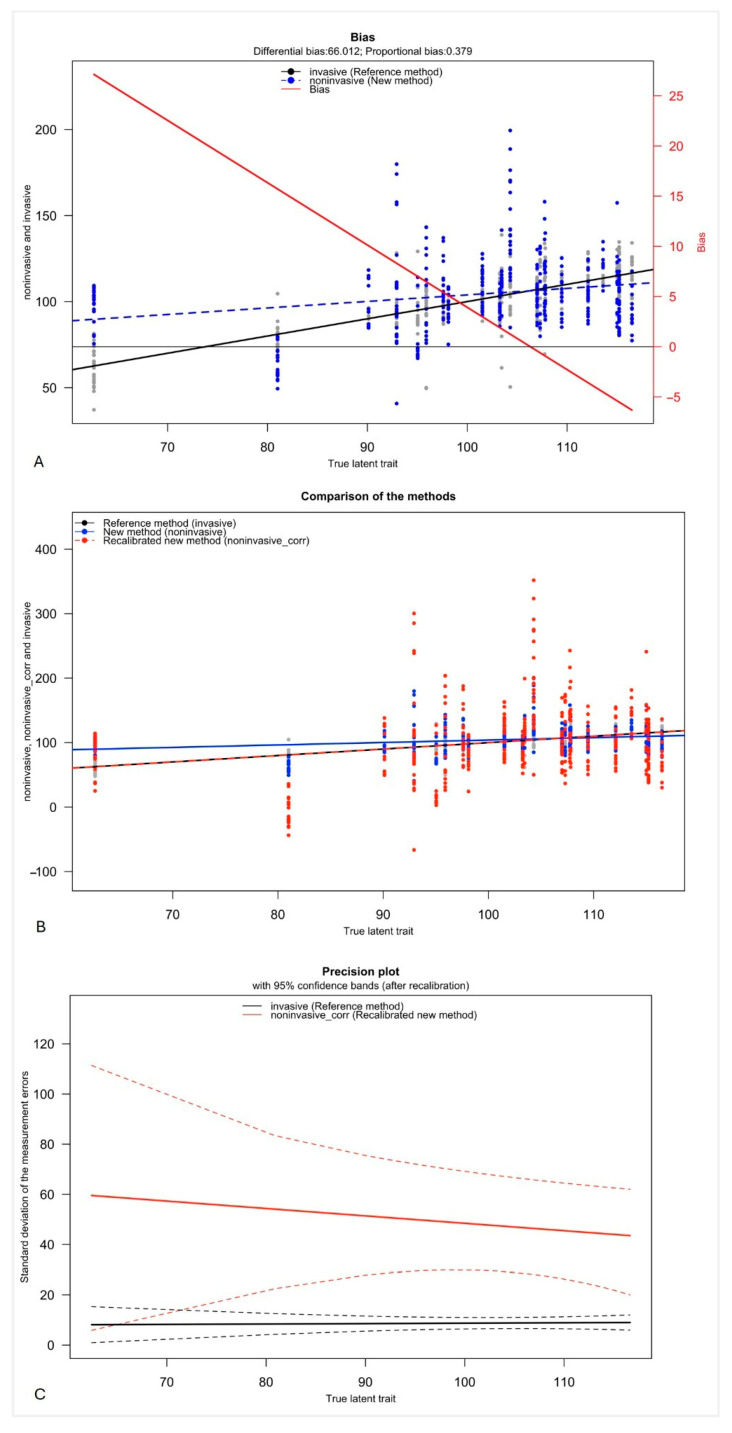
The Taffé plots comparing the non-invasively measured mean blood pressure with the invasive reference method. Bias plot (**A**): The plot shows the estimated bias (difference between the mBP measured non-invasively by fingertip vs. invasively) as a function (given as true latent trait). The solid black line depicts the reference method’s measurement over the “true latent trait” (*x*-axis, given in mmHg). The dashed blue line shows the new method. The red line indicates the bias (values are given on the right *x*-axis). Comparison plot (**B**): This plot shows the relationship between the measurements retrieved non-invasively and invasively as a reference method with a regression line. Precision plot (**C**): This plot illustrates the variability of the new method relative to the reference method, displaying the standard deviation of the methods with their confidence intervals.

The Taffé analysis was performed to further assess the systematic bias structure between the values retrieved non-invasively and the invasive values as a reference method. Figure 3A visualizes the systematic difference between the two methods with a differential bias of 66.012. The proportional bias of 0.379 implies that the bias is not constant but changes depending on the magnitude of the true latent trait. The downward slope of the bias visualizes the decrease in bias with an increase in the true latent trait, meaning that the non-invasive method overestimates the mBP at low values and underestimates the mBP at higher values.

The comparison plot reinforces the observations from the bias plot, demonstrating a directionally dependent error with a non-uniform proportional pattern (Figure 3B). The recalibration improves the overall alignment with the invasive reference, but the systematic deviations persist, particularly at lower values.

The precision plot further demonstrates that the invasive measurement provides superior precision, characterized by a low and relatively constant standard deviation of measurement errors across the full mBP range. This indicates stable and reliable estimates irrespective of the underlying latent trait. In contrast, the values obtained non-invasively display a wider dispersion, with overall variability markedly greater than that observed for the reference method (Figure 3C).

## 4. Discussion

This is a unique post hoc analysis of the accuracy of simultaneous assessment of the mBP obtained invasively with an intra-arterial line and non-invasively using the volume-clamp method with a finger cuff, using a large dataset of 385 paired, continuous mBP readings collected at rest and during stepwise cycling exercise in patients with PH. Using the Bland–Altman method, we observed only a small mean bias between the non-invasive finger-cuff measurements and the reference method. According to the Association for the Advancement of Medical Instrumentation, a BP-measuring device is considered acceptable if the measuring error is ≤10 mmHg, which is formally met in our data [16]. However, the limits of agreement were wide, and the bias was non-constant, although the non-invasive beat-to-beat BP measurement tended to overestimate BP at lower pressures and underestimate it at higher pressures compared to the invasive BP measurement. In this population with PH, the finger-cuff device provided reasonable average accuracy, but its variability with wide limits of agreement and non-constant bias means that the individual readings may deviate substantially from the intra-arterial values.

The previous studies and meta-analyses comparing finger-cuff devices to invasive arterial BP measurement have demonstrated that the volume clamp-based systems can provide reasonable estimates of the mBP and track hemodynamic changes, but do not consistently meet the criteria for clinical interchangeability in surgical or critically ill patients [3,5,6,7]. Although the underlying measurement principle is the same, the studies cited evaluated devices that were different from the one used in our investigation. Nonetheless, our findings align with these previous validation studies, which collectively indicate that single-point finger-cuff-based measurements have limited reliability for individual patient management.

The data on the performance of the finger-cuff method in patients with PH are limited, but the pathophysiology of PH suggests a substantial impact on measurement accuracy. The pulse contour algorithm underlying this method assumes aortic compliance based on the population averages; however, vascular stiffening and reduced compliance are common in patients with PH. This mismatch means the model does not accurately reflect the true pressure–volume relationship [17]. In addition, the volume-clamp technique has been shown to be highly sensitive to peripheral perfusion, vascular compliance, and edema—especially in patients with PH and right heart failure, where these factors are frequently altered [1,4].

Due to the non-uniform, directionally dependent error shown by the Taffé method, the calculation of a uniform correction is not advisable. The error pattern indicates that a single linear correction factor would not adequately address the proportional bias across the full mBP range. As the error varies depending on the level of the true latent trait, applying a uniform correction would risk introducing further inaccuracies. Even though a non-linear recalibration, as given by the Taffé method, could improve consistency and error structure by correcting the systematic bias and stabilizing the measurement variability, this would only improve the group-level accuracy, and, taking the wide limits of agreement into account, the individual readings would still deviate too much for precise clinical use.

Regarding the conflicting data, medical societies like the American Heart Association already recommend against using beat-to-beat BP values from finger monitoring for the diagnosis of hypertension or the management of patients [1]. We acknowledge that although the trending ability appears generally acceptable, the absolute values should be interpreted with caution. In PH, where peripheral vasoconstriction and edema are common, these limitations are particularly pronounced. The larger, disease-specific validation trials may help to verify our findings.

From a practical point of view, the findings of this study inform how continuous non-invasive BP monitoring should be integrated into clinical workflows for patients with PH. Although trending, the ability of a device to track directional changes in BP over time may still hold value for detecting gross hemodynamic deterioration during exercise; the substantial individual-level variability observed here precludes its use as a substitute for invasive monitoring in situations where precise BP values influence treatment decisions. This distinction is clinically relevant: reliance on non-invasive finger-cuff readings to titrate vasoactive medication, assess fluid responsiveness, or determine exercise limits in PH patients could lead to inappropriate management if the device systematically overestimates BP at lower pressures or underestimates it at higher pressures, as demonstrated by the Taffé analysis in this cohort.

Furthermore, the limitations of this study should be noted. The post hoc and single-center nature of the analysis and the relatively small sample of 24 patients limit the generalizability of the findings to other PH subgroups or care settings. The semi-supine cycling position, while clinically representative for this patient population, differs from upright exercise, and the device performance may differ further under different body positions or exercise modalities. Future studies using prospective designs with larger PH cohorts should provide stronger evidence for or against the clinical deployment of non-invasive continuous BP monitoring in this population.

## 5. Conclusions

In this study comparing invasive and non-invasive measurements of the mean blood pressure in PH, the small mean bias observed across all workloads suggests an acceptable agreement between the two methods at the group level, both at rest and during exercise. However, the wide dispersion of individual data points indicates limited accuracy at the individual level, meaning that single non-invasive measurements should be interpreted with caution. Therefore, although the device may be useful for monitoring trends, its clinical utility for precise hemodynamic assessment—particularly when guiding decision-making in patients with PH—appears limited.

## Figures and Tables

**Table 1:** The baseline characteristics. The data are presented as mean ± SD unless indicated otherwise. PAH, pulmonary arterial hypertension; CTEPH, chronic thromboembolic pulmonary hypertension; NT-proBNP, N-terminal pro B-type Natriuretic Peptide; SpO_2_, pulsoxymetric oxygen saturation.

Baseline Characteristics		Number (%) or Mean ± SD
No. of patients		24
Sex	Male	17 (71%)
	Female	7 (29%)
Age, yrs		59 ± 14
Body mass index, kg/m^2^		27.9 ± 4.6
Body surface area, m^2^		1.99 ± 0.19
Pulmonary hypertension classification	PAH	7 (29%)
	CTEPH	17 (71%)
WHO functional class	I	2 (8%)
	II	15 (63%)
	III	7 (29%)
NT-proBNP, ng/L (median [IQR])		258 [82, 485]
Heart rate, bpm		75 ± 11
SpO_2_, % (median [IQR])		93 [91, 95]
Mean pulmonary artery pressure, mmHg		37 ± 11
Pulmonary artery wedge pressure, mmHg		11 ± 2
Pulmonary vascular resistance, WU		5.2 ± 2.7

**Table 2:** The hemodynamic measurements. The data are presented as mean ± SD. *n* represents the number of valid data points included in the analysis.

Method	Mean Blood Pressure			
	Rest	*n*	End Exercise	*n*
invasive (arterial line)	96.1 ± 16.7 mmHg	77	106.8 ± 18.6 mmHg	35
non-invasive (finger cuff)	96.4 ± 17.2 mmHg	79	111.8 ± 21.6 mmHg	35

**Table 3:** The Bland–Altman test. The data are presented in mmHg. *n* represents the number of valid data points included in the analysis. LoA, limit of agreement.

	Bias	Lower LoA	Upper LoA	Spread	*n*
All data *	2.8	−39.6	45.3	84.9	385
Rest	0.8	−37.2	38.7	75.9	77
50% of max. workload	2.5	−41.3	46.3	87.6	30
Max. workload	4.8	−43.0	52.6	95.6	34

* includes all the paired mBP values at rest and during all exercise phases.

## Data Availability

The data are available upon request.

## Abbreviations

The following abbreviations have been used in this manuscript:

BP	Blood pressure
CTEPH	Chronic thromboembolic pulmonary hypertension
LoA	Limits of agreement
mBP	Mean blood pressure
PAH	Pulmonary arterial hypertension
PH	Pulmonary hypertension
